# Coxa Vara Treated by Division of the Femur below the Trochanters

**Published:** 1899-02-25

**Authors:** 


### COXA VARA TREATED BY DIVISION OF THE FEMUR BELOW THE TROCHANTERS.

At the last clinical meeting of the Medical Society Mr. Watson Cheyne showed cases illustrating the benefits to be attained in cases of coxa vara by dividing the femur below the trochanters and thus correcting the eversion of the leg. His idea in adopting this measure was to bring the foot and leg into the proper position so as to enable the patient to walk, leaving the hip-joint untouched. At the time the first case was done the term coxa vara had not been introduced, and the case was described as one of "external rotation of the legs." Apart from the restored usefulness of the limbs, a most remarkable and unexpected result of the operation had been that the progress of the deformity in the neck of the bone had been arrested. The operation consisted of an incision in the outer side of the upper part of the thigh, exposing and clearing the femur, dividing the femur transversely by a saw a little below the trochanters, forcible inversion of the foot and leg until the normal degree of complete inversion was obtained, and fixing the femur in the new position by means of a perforated aluminium plate placed over the line of division, and nailed to the two fragments by tin-tacks. The object of the operation was fully attained. The legs were in perfect position, inversion and eversion of the feet and legs was normal, and the legs were as useful as if nothing had been the matter with them. About three years after the operation in the first case an abscess had occurred which closed when the plate and tacks which were found loose in it were removed. In the second case the plate still remained. The deformity on the side operated on has in each case come to a standstill, while on the other side it has progressed.

